# Laparoscopic versus open gastrectomy for gastric cancer

**DOI:** 10.1186/s12957-020-1795-1

**Published:** 2020-01-27

**Authors:** Furong Zeng, Lang Chen, Mengting Liao, Bin Chen, Jing Long, Wei Wu, Guangtong Deng

**Affiliations:** 1 Xiangya hospital, Central South University, Changsha, China; 2 Taoyuan People’s Hospital, Taoyuan, Changde, China

**Keywords:** Gastric cancer, Meta-analysis, Recurrence, Mortality, Laparoscopic gastrectomy (LG), Open gastrectomy (OG)

## Abstract

**Background:**

Compared with open gastrectomy (OG), laparoscopic gastrectomy (LG) for gastric cancer has achieved rapid development and popularities in the past decades. However, lack of comprehensive analysis in long-term oncological outcomes such as recurrence and mortality hinder its full support as a valid procedure. Therefore, there are still debates on whether one of these options is superior.

**Aim:**

To evaluate the primary and secondary outcomes of laparoscopic versus open gastrectomy for gastric cancer patients

**Methods:**

Two authors independently extracted study data. Risk ratio (RR) with 95% confidence interval (CI) was calculated for binary outcomes, mean difference (MD) or the standardized mean difference (SMD) with 95% CI for continuous outcomes, and the hazard ratio (HR) for time-to-event outcomes. Review Manager 5.3 and STATA software were used for the meta-analysis.

**Results:**

Seventeen randomized controlled trials (RCTs) involving 5204 participants were included in this meta-analysis. There were no differences in the primary outcomes including the number of lymph nodes harvested during operation, severe complications, short-term and long-term recurrence, and mortality. As for secondary outcomes, compared with the OG group, longer operative time was required for patients in the LG group (MD = 58.80 min, 95% CI = [45.80, 71.81], *P* < 0.001), but there were less intraoperative blood loss (MD = − 54.93 ml, 95% CI = [− 81.60, − 28.26], *P* < 0.001), less analgesic administration (frequency: MD = − 1.73, 95% CI = [− 2.21, − 1.24], *P* < 0.001; duration: MD = − 1.26 days, 95% CI = [− 1.40, − 1.12], *P* < 0.001), shorter hospital stay (MD = − 1.37 days, 95% CI = [− 2.05, − 0.70], *P* < 0.001), shorter time to first flatus (MD = − 0.58 days, 95% CI = [− 0.79, − 0.37], *P* < 0.001), ambulation (MD = − 0.50 days, 95% CI = [− 0.90, − 0.09], *P* = 0.02) and oral intake (MD = − 0.64 days, 95% CI = [− 1.24, − 0.03], *P* < 0.04), and less total complications (RR = 0.81, 95% CI = [0.71, 0.93], *P* = 0.003) in the OG group. There was no difference in blood transfusions (number, quantity) between these two groups. Subgroup analysis, sensitivity analysis, and the adjustment of Duval’s trim and fill methods for publication bias did not change the conclusions.

**Conclusion:**

LG was comparable to OG in the primary outcomes and had some advantages in secondary outcomes for gastric cancer patients. LG is superior to OG for gastric cancer patients.

## Introduction

Gastric cancer is the third leading cause of cancer death and the fifth most common cancer worldwide [1–3]. Even though there is a steady decline in its incidence and mortality in recent years, an estimated 1,000,000 patients were newly diagnosed and more than 783,000 patients died from gastric cancer in 2018 [1]. More seriously, this trend has shown signs of change. A recent study demonstrated that the increasing rates of gastric cancer among people less than 50 years old might reverse the overall decline in the incidence of gastric cancer [4, 5].

Open gastrectomy (OG) remains the mainstay of curative approach for gastric cancer for a long time. Until 1994, Kitano firstly described the efficacy of laparoscopy gastrectomy (LG) in the case of early stage carcinoma in the antrum of the stomach [6]. Then, the employment of LG for gastric cancer has achieved rapid development and popularities in past decades due to minimal invasion, less blood loss, less time of using analgesic requirement and quicker recovery [7–10]. Another benefit of laparoscopic surgery is the capacity to observe the surgical field in a magnified view, which could help surgeons with more meticulous dissection of lymph nodes which is important to patient’s prognosis [11]. However, previous studies showed decreased number of harvested lymph nodes for gastric patients during LG compared with OG [12, 13]. Besides, like all the laparoscopic procedure, port site metastases and seeding during LG were inevitable because of intra-abdominal hyperpressure and adherence of laparoscopic instrument [14–17]. What is more, though there are some studies comparing the secondary outcomes between the LG and OG groups, lack of long-term oncological outcomes such as recurrence and mortality hinders its full support as a valid procedure [18–20]. Therefore, debates still exist whether LG is superior to OG for gastric cancer patients.

The aim of this meta-analysis was to identify and analyze random controlled trials (RCTs) in order to compare the primary and secondary outcomes of LG versus OG. Subgroup analyses were conducted to evaluate the primary outcomes which are key surgical and prognostic outcomes and may be influenced by the tumor stage and the gastrectomy type. Sensitivity analysis was implemented to validate the stability of the conclusion based on different effect models.

## Methods

### Search strategy

Two authors independently searched Pubmed, Embase, Cochrane Library, WANFANG, and China National Knowledge Internet until Nov. 25, 2018. The following combined search terms were used: (“Abdominal neoplasms” OR “Intestinal neoplasms” OR “Stomach neoplasms”) AND “Laparoscopy” AND “Gastrectomy” AND “Clinical trials” [21]. Details of the search strategies can be found in Additional file 1: Table S1.

### Selection criteria

Studies were selected based on the following inclusion criteria: (1) study design, RCT in English or Chinese (animal studies, observational studies, basic research, retrospective studies, case-control studies, quasi-randomized studies, case reports, and cohort studies were excluded); (2) participants, gastric cancer patients undergoing gastrectomy; (3) interventions, surgical operation comparing LG with OG; and (4) outcomes, primary outcomes and secondary outcomes. Primary outcomes are (1) number of lymph nodes harvested during surgery, (2) severe complications, (3) short-term and long-term recurrence, and (4) short-term and long-term mortality. Secondary outcomes are (5) operative time, (6) intraoperative blood loss, (7) measures of earlier postoperative recovery (analgesic administration, time to first flatus, first ambulation and first oral intake, hospital stay), (8) blood transfusion (number, quantity), and (9) total complications. If there were two or more studies from the same authors or institutions, only the study with the largest sample size was chosen. Studies were excluded if full text of the trial was not available or they did not fulfill the inclusion criteria.

### Data extraction and quality assessment

The records from the initial search were scanned by two authors to exclude any duplicate and irrelevant studies. The following data were extracted: first authors, publication date, country of origin, study period, tumor stage, gastrectomy type, lymph-node dissection, number of OG and LG cases, characteristics of the study population (including sex, age), follow-up, and primary and secondary outcomes (number of lymph nodes harvested during surgery, severe complications, recurrence and mortality; operative time, blood loss, indictors of earlier postoperative recovery (analgesic administration, first flatus, first ambulation, oral intake, hospital stay), blood transfusion (number, quantity), and total complications). Any discrepancies were resolved by discussion. Study quality was estimated using an adaptation of the Cochrane Handbook for Systematic Reviews of Interventions via the following characteristics: random sequence generation, allocation concealment, blinding of participants and personnel, blinding of outcome assessment, incomplete outcome data, selective data, and other bias.

### Statistical analysis

*I*^2^ and *P* value were used to evaluate the statistical heterogeneity. A fixed effects model was adopted with significant heterogeneity (*I*^2^ ≤ 50% and *P* ≥ 0.1), while a random effects model was employed in all other instances (*I*^2^ > 50% or *P* < 0.1) [22–24]. Risk ratio (RR) with 95% confidence interval (CI) was calculated for binary outcomes, mean difference (MD), or the standardized mean difference (SMD) with 95% CI for continuous outcomes and the hazard ratio (HR) for time-to-event outcomes. Subgroup analyses based on tumor stage and the type of gastrectomy were performed to evaluate the primary outcomes. Sensitivity analysis was used to explore the consistence of the conclusion based on fixed/random-effect models. Publication bias was evaluated by Egger’s test. If publication bias was conformed, the Duval’s trim and fill method was implemented to adjust for this bias. All statistical calculations were performed by Review Manager 5.3 (Cochrane collaboration. Copenhagen) and STATA software (Version 12.0; STATA Corporation, College Station, TX, USA). *P* value less than 0.05 was considered statistically significant.

## Results

### Search results and studies characteristics

**Fig. 1:**
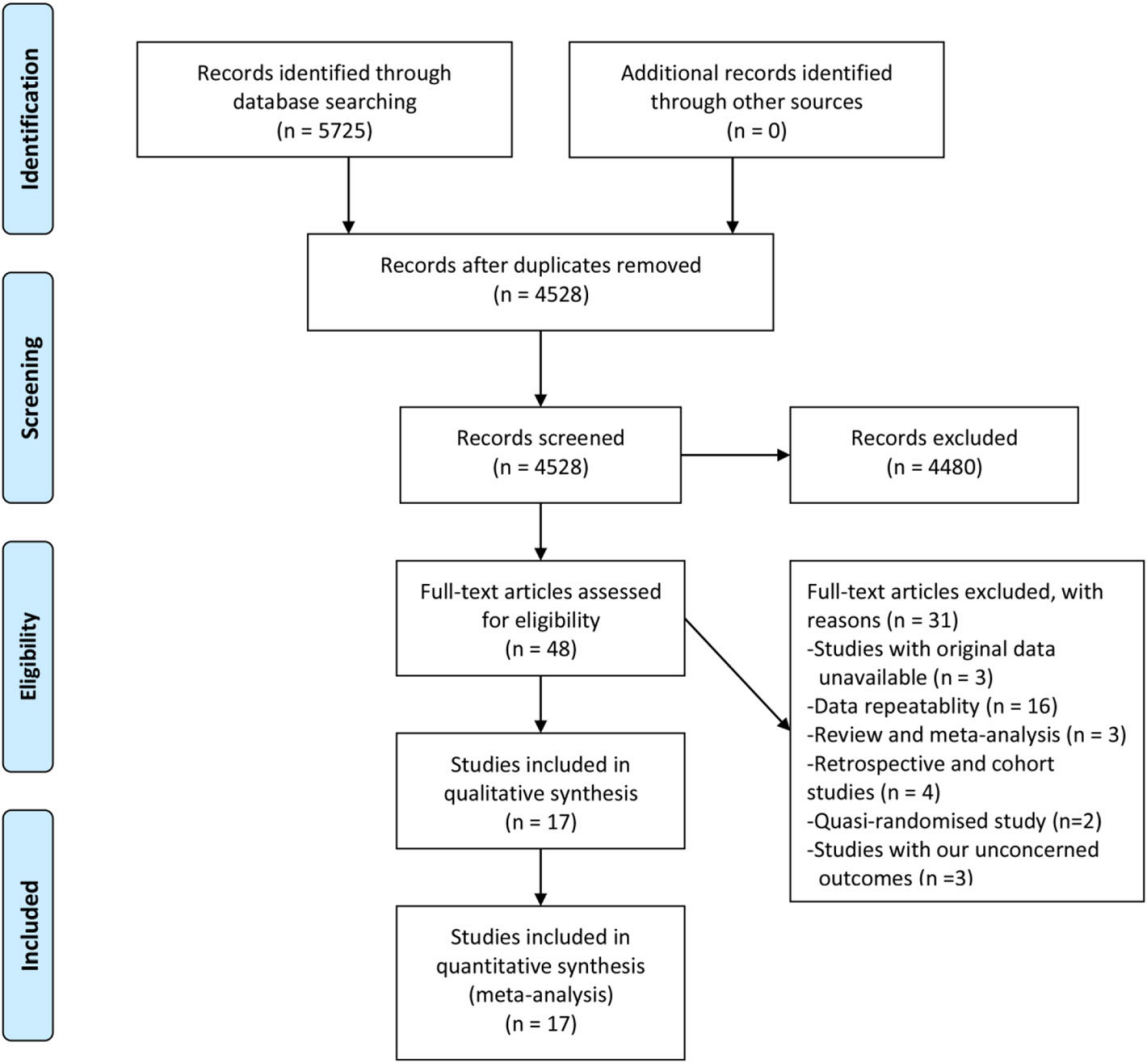
Flowchart of literature search and study selection process

Our search initially yielded 5725 studies with 1197 studies subsequently excluded due to duplication. After a review of the titles and abstracts, we obtained 48 studies by excluding an additional 4480 studies. We further excluded 31 studies by scanning the full text (original data unavailable [*n* = 3], data repeatability [*n* = 8], review and meta-analysis [*n* = 11], retrospective and cohort studies [*n* = 4], quasi-randomized studies [*n* = 2], and studies with our unconcerned outcomes [n = 3]). Finally, seventeen RCTs were included in our analysis [11, 25–40] (Fig. 1).

**Fig. 2:**
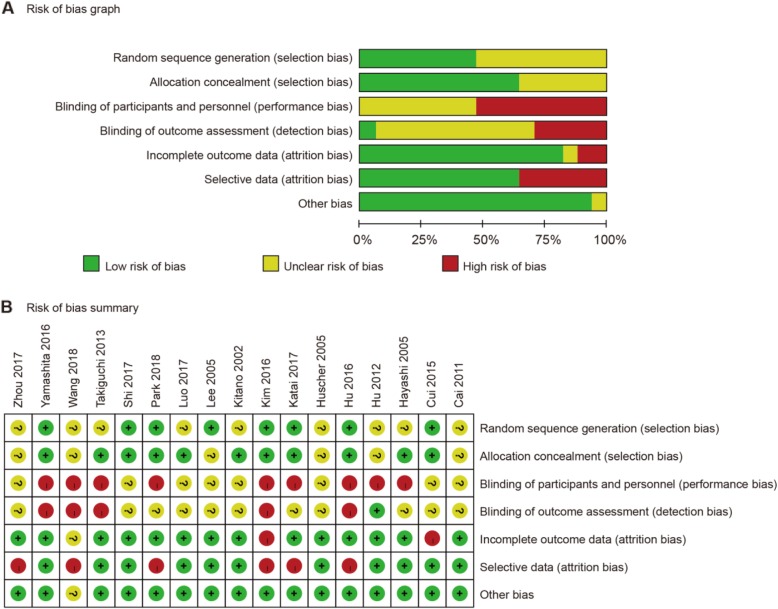
Risk of bias. **a** Risk of bias graph. **b** Risk of bias summary

Characteristics of seventeen eligible RCTs were presented in Table 1. These RCTs were published between 2002 and 2018, involving 5204 patients (50.3% patients with LG). There were no differences in the demographics and clinicopathological characteristics of patients in the LG and OG group for each study. Eight trials were conducted in China [25–27, 29, 35, 37, 39, 40], five studies in Japan [28, 31, 32, 36, 38], three in Korea [11, 33, 34], and one in Italy [30]. Early gastric cancer (EGC) patients were included in six studies [28, 32, 33, 36, 38, 39], and advanced gastric cancer (AGC) patients were enrolled in another six trials [25, 29, 34, 35, 37, 40]. Distal gastrectomy was adopted in nine trials [26, 28, 30, 32–34, 36, 38, 40]. The results of methodological quality assessment about each risk of bias item for each included trial were shown in Fig. 2.

**Table 1:** Baseline characteristics of studies included in the meta-analysis

Author year	Country	Study period	Tumor stage	Gastrectomy type	LND	Group	Cases	Age	M/F	Follow-up (months)
Kitano 2002 [32]	Japan	1998.11–2001.3	EGC	DG	NA	LG OG	14 14	63.2 60.1	9/5 8/4	24.3 18.8
Hayashi 2005 [28]	Japan	1999.12–2001.11	EGC	DG	D1	LG OG	14 14	56 62	9/4 13/1	39 45
Huscher 2005 [30]	Italy	1992.11–1996.2	EGC, AGC	DG	D1, D2	LG OG	30 29	63.2 63.6	18/12 21/8	52.2 49.7
Lee 2005 [33]	Korea	2001.11–2003.8	EGC	DG	D2, DSL	LG OG	24 23	56.6 59.5	11/13 15/8	14 14
Cai 2011 [25]	China	2008.3–2009.12	AGC	PG, DG, TG	D2	LG OG	49 47	60.2 60.3	39/10 37/10	22.1 22.1
Hu 2012 [26]	China	2009.1–2011.5	EGC, AGC	DG	NA	LG OG	41 41	60.9 64.3	20/21 21/20	1 1
Takiguchi 2013 [36]	Japan	2003.7–2006.1	EGC	DG	D1	LG OG	20 20	61.5 62.5	12/8 13/7	60 60
Cui 2015 [27]	China	2010.11–2012.9	EGC, AGC	PG, DG, TG	D2	LG OG	128 142	60.1 57.5	88/40 98/44	1 1
Hu 2016 [29]	China	2012.9–2014.12	AGC	DG, TG	D2	LG OG	519 520	56.5 55.8	380/139 346/174	1 1
Kim 2016 [11]	Korea	2006.2–2010.8	EGC, AGC	DG, TG	D1, D2	LG OG	644 612	56.8 57.8	425/219 412/200	1 1
Yamashita 2016 [38]	Japan	2005.11–2008.2	EGC	DG	DSL	LG OG	31 32	58 61	17/14 25/7	63 63
Luo 2017 [40]	China	2008.5–2012.4	AGC	DG	D2	LG OG	62 62	64.0 64.0	42/20 43/19	36 36
Zhou 2017 [39]	China	2012–2015	EGC	PG, DG, TG	D1, D2	LG OG	100 100	53.2 53.1	50/50 50/50	60 60
Shi 2017 [35]	China	2010.1–2012.6	AGC	PG, DG, TG	D2	LG OG	162 160	55.2 55	122/40 105/55	1 1
Katai 2017 [31]	Japan	2010.3–2013.11	EGC, AGC	DG, PPG	D1, D2	LG OG	457 455	63 64	289/173 275/184	1 1
Wang 2018 [37]	China	2014.3–2017.8	AGC	DG, TG	D2	LG OG	222 220	59.4 60.6	144/78 133/87	1 1
Park 2018 [34]	Korea	2010.6–2011.11	AGC	DG	D2	LG OG	100 96	58.6 60.1	69/31 65/31	38.2 38.2

*EGC* early gastric cancer, *AGC* advanced gastric cancer, *LND* lymph node dissection, *PG* proximal gastrectomy, *DG* distal gastrectomy, *TG* total gastrectomy, *PPG* pylorus preserving gastrectomy, *DSL* dissecting selected lymph nodes, *M* male, *F* female

### Primary outcomes

**Fig. 3:**
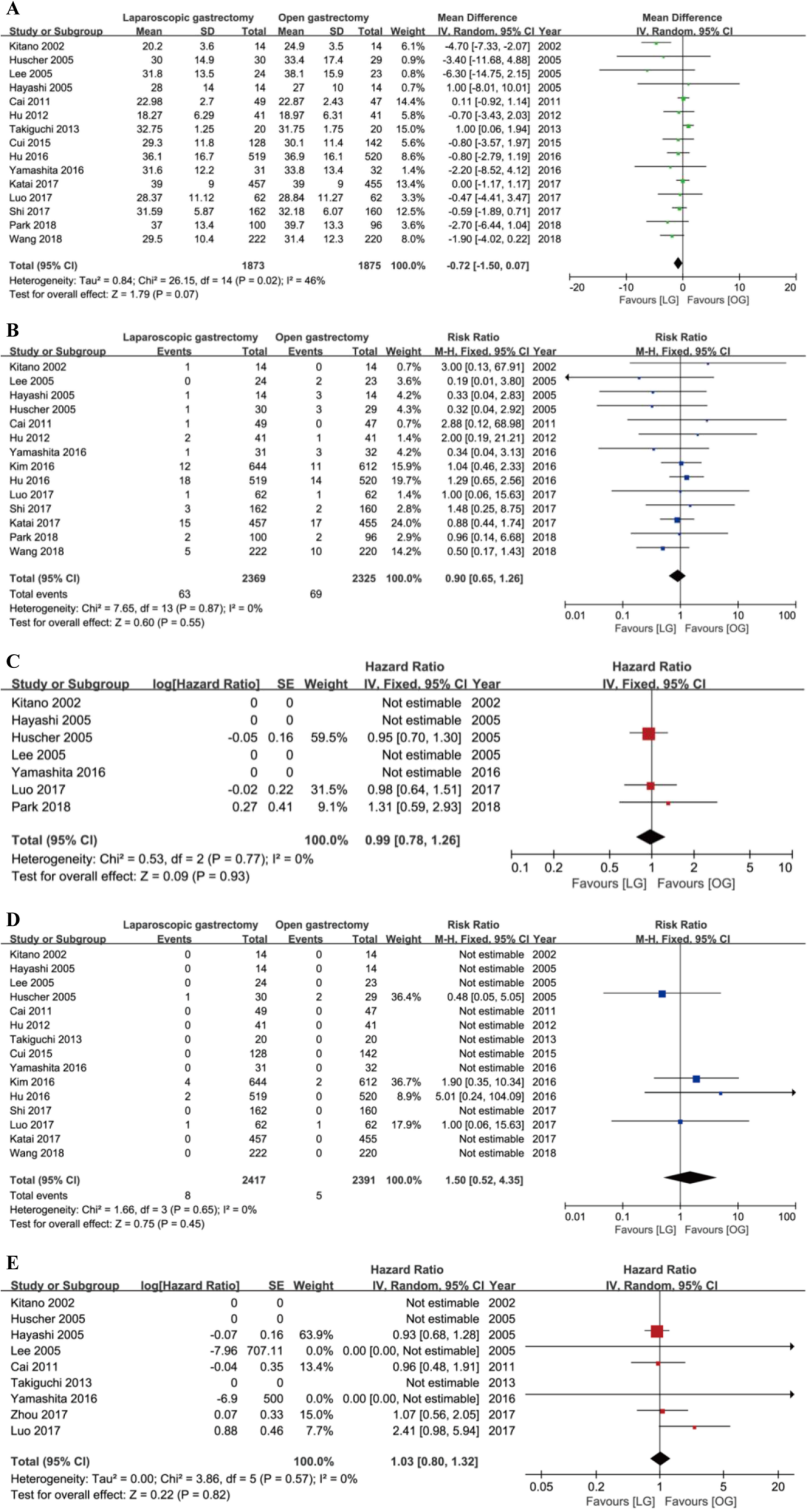
Forest plot between laparoscopy gastrectomy (LG) and open gastrectomy (OG) group on primary outcomes. **a** The number of lymph nodes harvested during surgery. **b** Severe complications. **c** Long-term recurrence. **d** Short-term mortality. **e** Long-term mortality

Sixteen trials reported the number of lymph nodes harvested during surgery. However, in Kim’s trial, the baseline was statistically significant in the extent of lymphadenectomy (*P* = 0.002). More patients suffered from D2 lymphadenectomy in the OG group than the LG group, which could cause a significant bias in the number of lymph nodes harvested during surgery [11]. Therefore, we excluded this trial in our analysis. Plotted data showed that there was no difference between these two groups in the number of lymph nodes harvested during surgery with a modern heterogeneity using the random model (MD = − 0.72, 95% CI = [− 1.50, 0.07], *P* = 0.07) (Fig. 3a).

Severe complications were defined when the extent of complications was up to grade III or more based on the Common Terminology Criteria for Adverse Events (CTCAE) ver. 4.0 or the Clavien-Dindo classification. Fourteen trials reported the severe complications. Fixed model showed no difference in these two groups without statistically significant heterogeneity (RR = 0.90, 95% CI = [0.65, 1.26], *P* = 0.55) (Fig. 3b).

Short-term recurrence was described as local recurrence, surgical recurrence, or distal metastases that existed within 6 months after surgery. Four trials reported the short-term recurrence while no patients were recurrent in the two groups. Therefore, we could conclude that there was no difference in the short-term recurrence between the LG and OG groups though we could not calculate the effect estimate. Seven trials reported the long-term recurrence which was defined as recurrence beyond 6 months after surgery. Fixed model showed no difference in these two groups without heterogeneity (HR = 0.99, 95% CI = [0.78, 1.26], *P* = 0.93) (Fig. 3c).

Fifteen trials reported short-term mortality which was regarded as death in hospital or within 1 month after surgery. Fixed model showed no difference in these two groups without statistically significant heterogeneity (RR = 1.50, 95% CI = [0.52, 4.35], *P* = 0.45) (Fig. 3d). Nine trials reported long-term mortality which was described as death out of hospital and beyond 1 month after operation. Fixed model showed no difference in these two groups without heterogeneity (HR = 1.03, 95% CI = [0.80, 1.32], *P* = 0.82) (Fig. 3e).

### Secondary outcomes

**Fig. 4:**
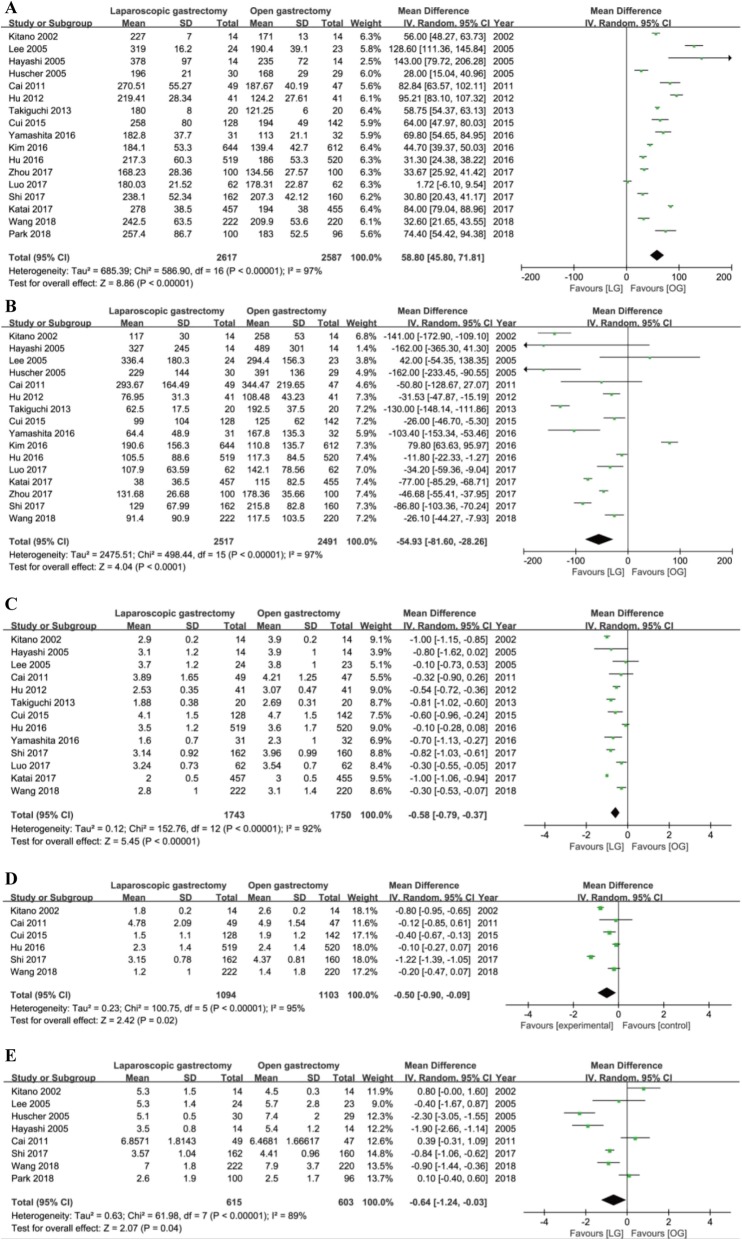
Forest plot between the LG and OG group on secondary outcomes. **a** Operative time. **b** Intraoperative blood loss on secondary outcomes. **c** Time to first flatus. **d** Time to first ambulation. **e** Time to first oral intake

**Fig. 5:**
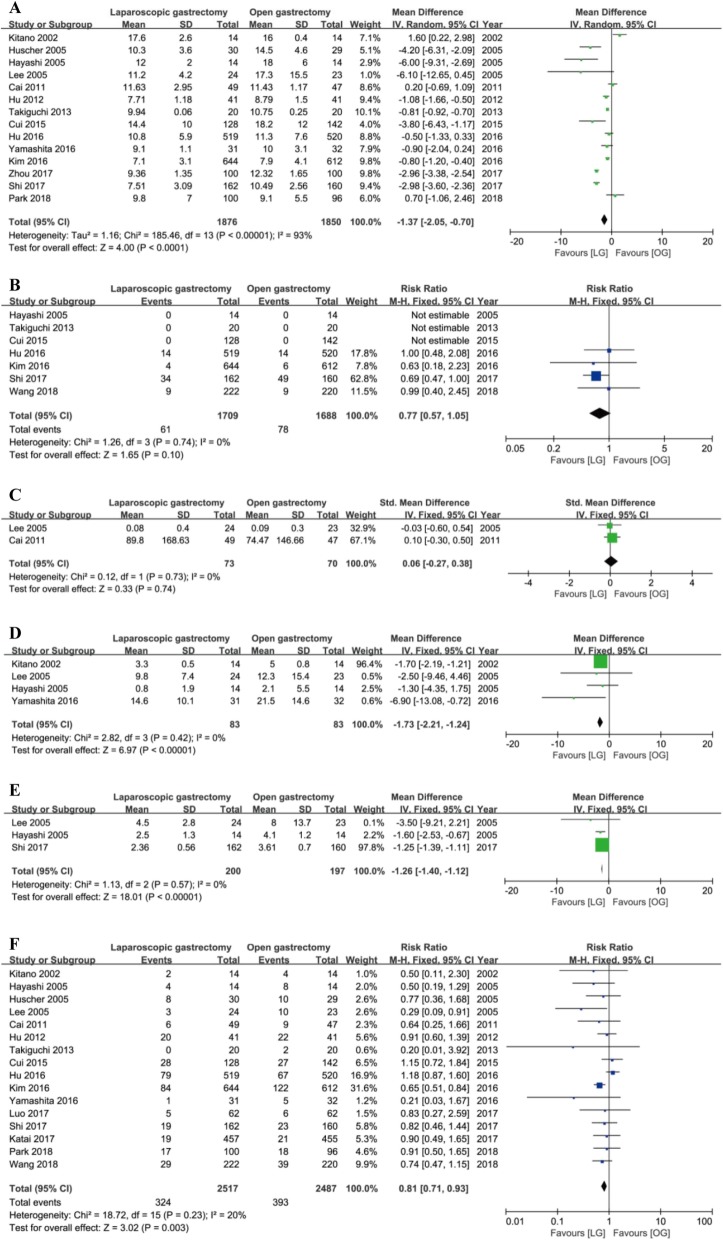
Forest plot between the LG and OG group on secondary outcomes. **a** Hospital stay. **b** The number of patients who need blood transfusion. **c** The quantity of blood transfusion. **d** The frequency of analgesic administration. **e** The duration of analgesic administration. **f** Total complications

There were longer operative time (MD = 58.80 min, 95% CI = [45.80, 71.81], *P* < 0.001), less intraoperative blood loss (MD = − 54.93 ml, 95% CI = [− 81.60, − 28.26], *P* < 0.001), less time to first flatus (MD = − 0.58 days, 95% CI = [− 0.79, − 0.37], *P* < 0.001), first ambulation (MD = − 0.50 days, 95% CI = [− 0.90, − 0.09], *P* = 0.02) and first oral intake (MD = − 0.64 days, 95% CI = [− 1.24, − 0.03], *P* < 0.04), and less hospital stay (MD = − 1.37 days, 95% CI = [− 2.05, − 0.70], *P* < 0.001) in the LG group versus the OG group with significant heterogeneity using random models (Fig. 4a–e**,** Fig. 5a).

There were no differences in the number of patients who need blood transfusion (RR = 0.77, 95% CI = [0.57, 1.05], *P* = 0.1) and the quantity of blood transfusion (SMD = 0.06, 95% CI = [− 0.27, 0. 38], *P* = 0.74) using a fixed model with no heterogeneity (Fig. 5b, c). Also, the fixed models showed that the frequency and the duration of analgesic administration was less and shorter in the LG group than the OG group with no heterogeneity (frequency: MD = − 1.73, 95% CI = [− 2.21, − 1.24], *P* < 0.001; I^2^ = 0, *P* = 0.42; duration: MD = − 1.26, 95% CI = [− 1.40, − 1.12], *P* < 0.001; *I*^2^ = 0, *P* = 0.57) (Fig. 5d, e).

Total complications were defined as complications that occurred during the same hospitalization or within 30 days after the operation. Sixteen trials reported the total complications. Fixed model showed that patients in the LG group underwent fewer total complications after surgery than the OG group (RR = 0.81, 95% CI = [0.71, 0.93], *P* = 0.003) without statistically significant heterogeneity (Fig. 5f).

### Subgroup analysis

Primary outcomes consist of lymph nodes harvested during surgery, severe complications, short and long-term recurrence, and mortality. Considering that primary outcomes are the key surgical and prognostic markers, we conducted the subgroup analysis about these indicators. Subgroup analysis was stratified based on the different cancer stages (early gastric cancer and advanced gastric cancer) and different types of gastrectomy (distal gastrectomy). Subgroup analysis showed no difference in lymph nodes harvested during surgery, severe complications, recurrence, and mortality between these two groups. Detailed results were shown in Tables 2 and 3.

**Table 2:** Subgroup analysis of laparoscopic versus open gastrectomy stratified by different tumor stage

Outcome	Studies	Participants	Heterogeneity		Model	WMD, RR, or HR	95% CI	P
			*I*^2^	*P*				
Lymph nodes harvested								
1. EGC	5	206	79%	< 0.001	Random	− 2.02	[− 5.76, 1.72]	0.29
2. AGC	6	2219	0	0.49	Fixed	− 0.51	[− 1.19, 0.18]	0.15
Severe adverse complications								
1. EGC	4	166	0	0.60	Fixed	0.44	[0.14, 1.39]	0.16
2. AGC	6	2219	0	0.73	Fixed	1.03	[0.62, 1.69]	0.92
Short-term recurrence								
1. EGC	4	166	Totals not selected					
2. AGC	0	0						
Long-term recurrence								
1. EGC	4	166	Totals not selected					
2. AGC	2	320	0	0.53	Fixed	1.05	[0.72, 1.53]	0.82
Short-term mortality								
1. EGC	5	206	Totals not selected					
2. AGC	5	2023	0	0.44	Fixed	2.34	[0.35, 15.70]	0.38
Long-term mortality								
1. EGC	6	406	0	0.99	Fixed	0.96	[0.72, 1.27]	0.76
2. AGC	2	220	61%	0.11	Random	1.45	[0.59, 3.55]	0.42

**Table 3:** Subgroup analysis of laparoscopic versus open gastrectomy stratified by different type of gastrectomy

Outcome	Studies	Participants	Heterogeneity		Model	WMD, RR, or HR	95% CI	P
			I^2^	P				
Lymph nodes harvested								
1. Distal gastrectomy	9	667	64%	0.005	Random	−1.64	[−3.76, 0.39]	0.11
Severe adverse complications								
1. Distal gastrectomy	8	627	0	0.81	Fixed	0.62	[0.29, 1.34]	0.22
Short-term recurrence								
1. Distal gastrectomy	4	166	Totals not selected					
Long-term recurrence								
1. Distal gastrectomy	7	545	0	0.77	Fixed	0.99	[0.78, 1.26]	0.93
Short-term mortality								
1. Distal gastrectomy	8	471	0	0.69	Fixed	0.65	[0.11, 3.79]	0.64
Long-term mortality								
1. Distal gastrectomy	7	389	0	0.28	Fixed	1.22	[0.68, 2.17]	0.50

### Sensitivity analysis and publication bias

**Fig. 6:**
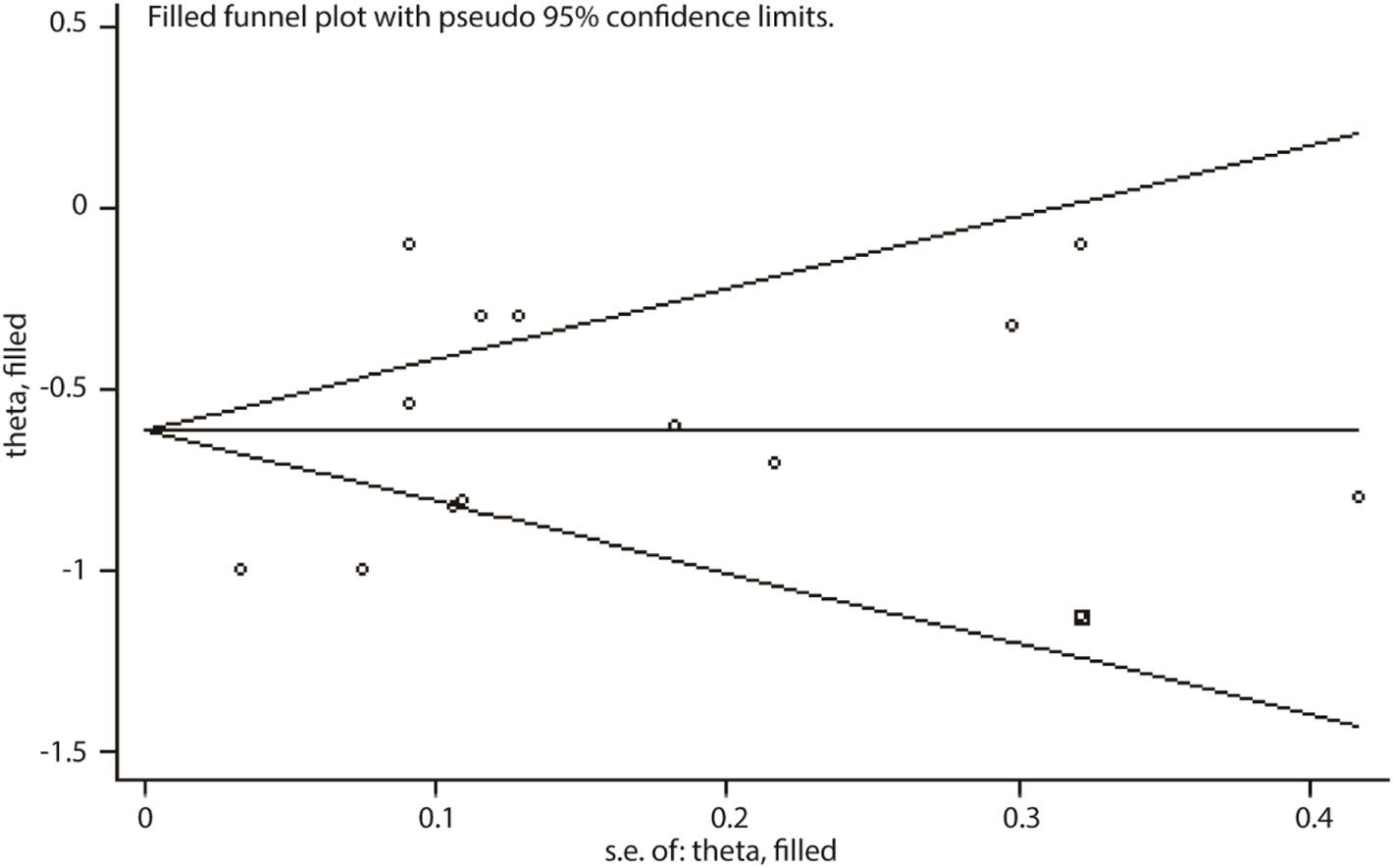
Filled funnel plot with pseudo 95% confidence limits on time to first flatus

Sensitivity analysis is an analytic procedure which could be used to explore the source of uncertainty in the pooled results. We used fixed/random-effect models to test each comparison and arrived at a consistent conclusion (data not shown). Egger’s test was conducted for each comparison to evaluate the publication bias. There exists publication bias in the number of lymph nodes harvested during surgery, the duration of analgesic administration and the time to first flatus (Table 4); however, when applying the trim-and-fill method, there were not any trials trimmed in the number of lymph nodes harvested and the duration of analgesic administration. About the time to first flatus, after filling one trial, the revised result was still consistent using random model (MD = − 0.61 days, 95% CI = [− 0.82, − 0.41], *P* < 0.001) or fixed model (MD = − 0.81 days, 95% CI = [− 0.86, − 0.76], *P* < 0.001), indicating no publication bias in the comparison. The filled plot was shown in Fig. 6.

**Table 4:** Publication bias by Egger’s test

Outcome	Studies	*P* (Egger’s test)
Operative time	17	0.75
Blood loss	16	0.82
Blood transfusion		
1. Number	7	0.49
2. Quantity	2	–
Lymph nodes harvested	16	0.02
Analgesic administration		
1. Frequency	4	0.42
2. Duration	3	0.03
Hospital stay	14	0.30
Time to first flatus	13	0.03
Time to first ambulation	6	0.53
Time to first oral intake	8	0.75
Adverse complications		
1. Total	16	0.10
2. Severe	14	0.52
Recurrence		
1. Short-term	4	–
2. Long-term	7	0.15
Mortality		
1. Short-term	15	0.97
2. Long-term	9	0.27

## Discussion

Though there are some meta-analyses comparing the safety and efficacy of the LG and OG for gastric cancer patients, there still exist some concerns about the number of lymph nodes harvested during the surgery and the long-term outcomes [12, 13, 18–20]. In our meta-analysis, we summarized the primary and secondary outcomes of LG versus OG for gastric cancer patients. After an extensive search of the literature, 17 RCTs were identified and included.

Of the primary outcomes, they are key surgical and prognostic indictors including the number of lymph nodes harvest during surgery, severe complications, recurrence, and mortality. As for the number of lymph nodes harvested during surgery, we excluded Kim’s trail because there was statistical significance in the extent of lymphadenectomy. There are 390 patients with D2 lymphadenectomy and 216 patients with D1 lymphadenectomy in the OG group while 360 and 284 patients suffered from D2 and D1 lymphadenectomy in the LG group, separately (*P* = 0.004). Kim et al. also admitted that this bias could be the reason that more lymph nodes were dissected in the OG group than in the LG group [11]. Therefore, it is necessary to exclude the trial in the pooled analysis of the number of lymph nodes dissection during surgery. Through the meta-analysis, the plotted data demonstrated that there were no statistically significant differences in primary outcomes between the LG and OG groups. Stratified by the different cancer stage and different types of gastrectomy, subgroup analysis was conducted to check the sensitivity and stability of the results. The conclusion was consistent, which suggested that LG has a comparable efficacy compared with OG for gastric cancer patients.

As for the secondary outcomes, they consist of operative time, intraoperative blood loss, blood transfusion (number, quantity), measures of earlier postoperative recovery (analgesic administration, time to first flatus, first ambulation and first oral intake, and hospital stay), and total complications. Plotted data showed that there were no differences between the two groups in the number of patients who need transfusions and the quantity of blood transfusions. Longer operative time was required for patients in the LG group than the OG group. However, compared with patients in OG group, patients in LG group lost less blood during operation, achieved lower total complications; required less analgesic administration; shorter time to first flatus, first ambulation, and first oral intake; and shorter hospital stay. That means LG has an advantage over OG in the safety for gastric cancer patients.

In order to check the stability of our results, we conducted sensitivity analysis. We used fixed/random models to test each comparison and the conclusions were unchanged. Egger’s test showed that publication bias existed in the number of lymph nodes harvested during surgery, the duration of analgesic administration and the time to first flatus. Conclusions were consistent by the Duval’s trim and fill method, which means our results were stable and reliable.

Despite all this, this meta-analysis has some limitations. Firstly, all these RCTs have high or unclear risk in blinding due to medical ethics. Secondly, heterogeneity exists in operative time, blood loss, analgesic administration, hospital stay, and time to first flatus, ambulation, and oral intake. Finally, limited data were available to compare the hospital costs and health-related quality of life which are also important for patients to choose the method of operation [26, 39, 40].

## Conclusion

In our analysis, we could conclude that LG was comparable to OG in the primary outcomes and had some advantages in secondary outcomes. That means LG is superior to OG for gastric cancer patients.

## Supplementary information

**Additional file 1:** **Table S1.** The detailed search strategies.

## Data Availability

All data generated or analyzed during this study are included in this published article.

## Abbreviations

AGC: Advanced gastric cancer
CI: Confidence interval
EGC: Early gastric cancer
HR: Hazard ratio
LG: Laparoscopic gastrectomy
MD: Mean difference
OG: Open gastrectomy
RCT: Randomized controlled trials
RR: Risk ratio
SMD: Standardized mean difference
