# Supplementary material for: Laparoscopic versus open gastrectomy for gastric cancer
Source: World J Surg Oncol. 2020 Jan 27;18:20. doi: 10.1186/s12957-020-1795-1 (PMC6986035; doi:10.1186/s12957-020-1795-1)
Supplement: Supplementary file 1 — Additional file 1: Table S1. The detailed search strategies. [file 12957_2020_1795_MOESM1_ESM.docx]

**Table S1. Search strategies.**

| Database | Search strategies |
| --- | --- |
| Pubmed | #1 carcin* or cancer* or neoplas* or tumour* or tumor* or cyst* or growth* or adenocarcin* or malig*  #2 Intestin* or Digest* or Gastr* or gut or epigastr* or stomach*  #3 #1 AND #2  #4 "Stomach Neoplasms"[Mesh] OR "Intestinal Neoplasms"[Mesh] OR "Abdominal Neoplasms"[Mesh]  #5 #3 OR #4  #6 "Laparoscopy"[Mesh]  #7 laparoscopy or laparoscopic  #8 #6 OR #7  #9 "Gastrectomy"[Mesh]  #10 Gastrectom*  #11 #9 OR #10  #12 #5 AND #8 AND #11  #13 ((clinical[tiab] AND trial[tiab]) OR "clinical trials as topic"[mesh] OR "clinical trial"[pt] OR random*[tiab] OR "random allocation"[mesh] OR "therapeutic use"[sh])  #14 #12 AND #13 |
| Embase | #1 carcin* or cancer* or neoplas* or tumour* or tumor* or cyst* or growth* or adenocarcin* or malig*  #2 Intestin* or Digest* or Gastr* or gut or epigastr* or stomach*  #3 #1 AND #2  #4 exp stomach tumor/ or exp abdominal tumor/ or exp stomach cancer/ or exp abdominal cancer/ or exp intestine cancer/ or exp stomach carcinoma/ or exp stomach carcinogenesis/ or exp stomach carcinoid/  #5 #3 OR #4  #6 exp laparoscopy/  #7 laparoscopy or laparoscopic  #8 #6 OR #7  #9 exp gastrectomy Billroth I/ or exp gastrectomy/ or exp partial gastrectomy/ or exp gastrectomy Billroth II/  #10 Gastrectom*  #11 #9 OR #10  #12 #5 AND #8 AND #11  #13 ('clinical':ti,ab AND 'trial':ti,ab) OR 'clinical trial'/exp OR random* OR 'drug therapy':lnk  #14 #12 AND #13 |
| Cochrane Library | #1 (carcin* or cancer* or neoplas* or tumour* or tumor* or cyst* or growth* or adenocarcin* or malig*)  #2 (Intestin* or Digest* or Gastr* or gut or epigastr* or stomach* or abdomin*)  #3 #1 or #2  #4 MeSH descriptor: [Abdominal Neoplasms] explode all trees  #5 MeSH descriptor: [Intestinal Neoplasms] explode all trees  #6 MeSH descriptor: [Stomach Neoplasms] explode all trees  #7 #4 or #5 or #6  #8 #3 or #7  #9 (laparoscopy or laparoscopic)  #10 MeSH descriptor: [Laparoscopy] explode all trees  #11 #9 or #10  #12 gastrectomy  #13 MeSH descriptor: [Gastrectomy] explode all trees  #14 #12 or #13  #15 #8 and #11 and #14 |
| WANFANG | Adjust based on the above strategy |
| China National Knowledge Internet | Adjust based on the above strategy |
